# The Role of Probiotics in Preventing Gestational Diabetes: An Umbrella Review

**DOI:** 10.3390/jcm14145168

**Published:** 2025-07-21

**Authors:** Simone Cosmai, Sara Morales Palomares, Cristina Chiari, Daniela Cattani, Stefano Mancin, Alberto Gibellato, Alessandra Valsecchi, Marco Sguanci, Fabio Petrelli, Giovanni Cangelosi, Diego Lopane, Beatrice Mazzoleni

**Affiliations:** 1 Department of Biomedical Sciences, Humanitas University, Via Rita Levi Montalcini 4, Pieve Emanuele, 20072 Milan, Italy; simone.cosmai@gavazzeni.it (S.C.); cristina.chiari@gavazzeni.it (C.C.); daniela.cattani@humanitas.it (D.C.); alberto.gibellato@gavazzeni.it (A.G.); alessandra.valsecchi@gavazzeni.it (A.V.); diego.lopane@hunimed.eu (D.L.);; 2 Department of Pharmacy, Health and Nutritional Sciences (DFSSN), University of Calabria, 87036 Rende, Italy; sara.morales@unical.it; 3 IRCCS Humanitas Research Hospital via Manzoni 56, 20089 Rozzano, Italy; 4 A.O. Polyclinic San Martino Hospital, Largo R. Benzi 10, 16132 Genova, Italy; sguancim@gmail.com; 5 School of Pharmacy, Experimental Medicine and “Stefania Scuri” Public Health Department, University of Camerino, 62032 Camerino, Italy; fabio.petrelli@unicam.it

**Keywords:** gestational diabetes, probiotics, prevention, umbrella review, public health

## Abstract

**Background/Objectives**: Gestational diabetes (GD), which affects approximately 15% of pregnancies worldwide, poses significant risks to both maternal and fetal health, underscoring the need for effective prevention and management strategies. This umbrella review aims to evaluate the role of probiotics in the prevention of GD. **Methods:** The review was conducted in accordance with the *Joanna Briggs Institute (JBI) Manual for Evidence Synthesis*. A comprehensive literature search was performed in November 2024 across four databases: PubMed/Medline, Cochrane Library, Embase, and CINAHL. A total of 307 articles were identified, of which 6 met the inclusion criteria and were included in the final synthesis. **Results:** Probiotic supplementation was associated with a significant reduction in the incidence of GD in selected populations, particularly in women with a body mass index (BMI) < 26, age < 30 years [Relative Risk (RR): 0.58], and *p* < 0.05 in the other studies included, alongside improvements in several metabolic parameters. However, consistent benefits on maternal or neonatal complications were not observed but a 33% reduction in GD was confirmed (RR 0.67). The combination of probiotics with healthy lifestyle behaviors appeared to exert a stronger protective effect against GD and its potential complications. **Conclusions:** This umbrella review suggests that probiotics—particularly multi-strain formulations—may have a potential role in reducing the risk of GD in certain populations. However, the findings across the included studies are inconsistent and sometimes conflicting. While probiotics are generally considered safe and have recognized benefits for metabolic health, their efficacy as an adjunct intervention for GD prevention remains not fully clear. Further well-designed research is needed to clarify which specific probiotic interventions may be effective and to better guide clinical practice.

## 1. Introduction

Gestational diabetes (GD) is defined as any degree of glucose intolerance with onset or first recognition during pregnancy. The optimal value of blood glucose concentration to diagnose GD remains controversial [1,2,3]. The prevalence of GD varies internationally and affects approximately 15% of pregnant women [3,4,5,6]. GD is typically detected between the 13th and 26th week of gestation, or early in the third trimester [7]. With the implementation of screening programs, GD is usually diagnosed before it becomes symptomatic. While many women remain asymptomatic, signs and symptoms associated with hyperglycemia, such as polyuria, polydipsia, blurred vision, and fatigue, may occur when GD is undetected or poorly controlled [8]. Universal screening between the 24th and 28th week of gestation is recommended, involving an oral glucose tolerance test (OGTT) with 75 g of glucose and an assessment of fasting, assessing blood glucose levels at 1 h, and 2 h. A single glucose measurement exceeding the threshold at any point during the OGTT is sufficient for diagnosis [9]. Maintaining normal blood glucose levels during pregnancy is essential to prevent short- and long-term adverse pregnancy outcomes [10]. GD increases maternal risks, including premature labor, cesarean delivery, hypertensive disorders, preeclampsia, and recurrent GD in future pregnancies [11]. Macrosomia (birth weight > 4000 g or 4500 g) is associated with maternal complications, such as perineal trauma and, more commonly, unplanned cesarean section due to failure to progress during labor, while uterine rupture is a rare occurrence [12]. Additionally, macrosomia can result in birth injuries, including shoulder dystocia, nerve paralysis, and fractures. GD is further associated with neonatal hypoglycemia (<45 mg/dL), hyperbilirubinemia (bilirubin levels approximately 2–3 mg/dL), polycythemia (hemoglobin > 16 g/dL), hypocalcemia (<8.8 mg/dL), and respiratory distress syndrome [10]. In the years following pregnancy, GD has been linked to an increased maternal risk of developing type 2 diabetes (T2D), cardiovascular disease, and metabolic syndrome. Offspring of mothers with GD are at greater risk of childhood overweight/obesity, cardiovascular diseases, developmental disorders, neurological impairment, and diabetes [13]. Neonates born to mothers with GD have higher risks of respiratory distress, jaundice, and hypoglycemia [14,15]. Long-term health impacts extend into childhood and adulthood, including obesity, diabetes, metabolic syndrome [16], and adverse neurodevelopmental outcomes [17,18]. Several risk factors contribute to the development of GD. Advanced maternal age, overweight (BMI ≥ 25 kg/m^2^), and obesity (BMI ≥ 30 kg/m^2^) are among the most common risk factors [19]. Additional risk factors include non-modifiable aspects such as ethnic background (e.g., Hispanic or Asian), previous GD diagnosis, history of delivering a macrosomic infant, family history of T2D, and conditions characterized by insulin resistance, such as polycystic ovary syndrome. Modifiable factors are categorized as pre-pregnancy (physical inactivity, poor dietary quality) and peri-pregnancy factors (excessive gestational weight gain). Poor maternal diet quality, characterized by low dietary fiber intake, consumption of foods with a high glycemic index, and high intake of sugar-sweetened beverages, increases GD risk [13]. Lower physical activity levels and increased sedentary behavior in early pregnancy also elevates GD risk [13]. Preventive interventions have been implemented before conception, during pregnancy, and inter-conceptionally. Approaches include dietary modifications, physical activity promotion, dietary supplementation, and pharmacological interventions. Dietary interventions typically involve increased fiber intake [20], low glycemic index diets [21], or broader healthy eating recommendations as part of comprehensive lifestyle interventions [22]. Interventions to promote physical activity include general advice or specific personalized programs, such as aerobic activities, static cycling, or yoga [23,24,25,26,27,28,29,30,31,32,33]. These interventions have been employed individually [24] or in combination with dietary modifications [26,27,28,29]. Dietary supplementation: Supplements such as probiotics have been studied [34,35,36] including, myo-inositol [37,38,39], vitamin D [40,41], and fish oil to prevent GD [42]. When normoglycemia is not achieved through lifestyle modifications, pharmacological treatment is required. In most countries, insulin remains the standard and most widely used option to achieve glycemic control [2,3,4]. However, metformin is also used in selected cases, depending on clinical guidelines and patient-specific considerations [43], and to prevent GD in pregnant women with a history of polycystic ovary syndrome [44]. Probiotics are defined by the Food and Agriculture Organization (FAO) and the World Health Organization (WHO) as “live microorganisms which, when administered in adequate amounts, confer a health benefit on the host” [45]. Regular probiotic consumption has demonstrated positive modulation of gut microbiota composition [46]. This umbrella review aims to summarize the existing secondary literature to clarify the role of probiotics in preventing GD.

## 2. Materials and Methods

### 2.1. Formulation of the Research Question

The research question for this study was developed using the PICO tool [47]. Three main aspects of the PICO strategy were included in this review: P = pregnant women and GD; I = probiotics; and O = prevention of GD.

### 2.2. Transparency and Search Strategy

An umbrella review was conducted following the Joanna Briggs Institute framework [48]. For transparency and reproducibility, the protocol was recorded to Open Science Framework (OSF): osf.io/4kw2f. For reporting, this review adhered to the preferred reporting items for overviews of reviews (PRIOR) statement (Supplementary Table S1: Check List) [49]. This literature review was guided by the following research question: what is the role of probiotics in preventing GD and possible complications?

The bibliographical search was carried out on 6 November 2024, by interrogating four biomedical databases, MEDLINE (PubMed), Cumulative Index to Nursing and Allied Health Literature (CINAHL), The Cochrane Library, and EMBASE using the keywords ‘gestational diabetes’, ‘probiotic supplementation’, ‘probiotics’, ‘probiotic agent’, ‘probiotic*’, appropriately combined using Boolean operators. The search strategies and filters used are described in Supplementary Table S2. Included studies addressed the research question and met the following criteria: full text availability and classification as secondary literature (systematic reviews or meta-analyses). No publication date restrictions were applied. Documents focusing on other GD preventive interventions, such as dietary interventions, physical exercise, or supplements like vitamin D, myo-inositol, and fish oil, were excluded. Two authors (C.C. and A.G.) independently screened all titles and abstracts retrieved from electronic searches, excluding duplicates and irrelevant records using Rayyan software (https://www.rayyan.ai/) (2025 version) (accessed on 1 May 2025). Conflicts were resolved by consulting a third author (C.S.). Subsequently, full-text articles were independently assessed for inclusion by the same two researchers (C.C. and A.G.). Any disagreements were resolved through consensus, with arbitration provided by a third expert (C.S.) who had not initially reviewed the articles. Relevant documents were summarized independently by each author using the data extraction table proposed by Aromataris et al. [50].

### 2.3. Inclusion and Exclusion Criteria

Studies were selected based on several inclusion criteria. Specifically, studies were required to directly address the research question, focus on probiotics for GD prevention, and consist exclusively of secondary literature (systematic reviews or meta-analyses). Studies written in languages other than English were eligible, with no geographical limitations. Conversely, exclusion criteria ruled out studies that did not address the research question. Primary studies, qualitative research, narrative reviews, and grey literature were excluded. Furthermore, articles involving obese women or those who already had diabetes or GD were excluded.

### 2.4. Evaluation of Risk of Bias and Methodological Quality of Studies

The risk of bias and methodological quality of the included articles were independently evaluated by two reviewers (C.C. and A.G.) using the critical appraisal checklist for systematic reviews and research syntheses from the Joanna Briggs Institute [48]. Disagreements were resolved by a third impartial reviewer (C.S.). The risk of bias in individual studies was assessed using the following criteria, based on a previous study [51]: a low risk of bias was assigned if 70% or more of the answers were “yes,” a moderate risk was assigned if 50% to 70% of the answers were “yes,” and a high risk of bias was assigned if less than 50% of the answers were “yes”.

### 2.5. Data Extraction and Synthesis

Data were extracted using a structured approach following the guidelines proposed by Aromataris et al. (2015) [50] and for a quality synthesis of the studies an included GRADE approach was performed [52]. To ensure consistency and accuracy, all extracted data were compiled into synthesis tables. In this review, while the benefits of meta-analysis are acknowledged, a combined quantitative synthesis was deemed not feasible due to the heterogeneity of the included studies. This variability, characterized by differences in intervention types and methodologies for quantifying relationships between variables, led to inconsistencies in both the methodological and statistical approaches. As a result, a detailed narrative synthesis was chosen, following established guidelines for synthesis without meta-analysis (SWiM) [53].

## 3. Results

**Figure 1:**
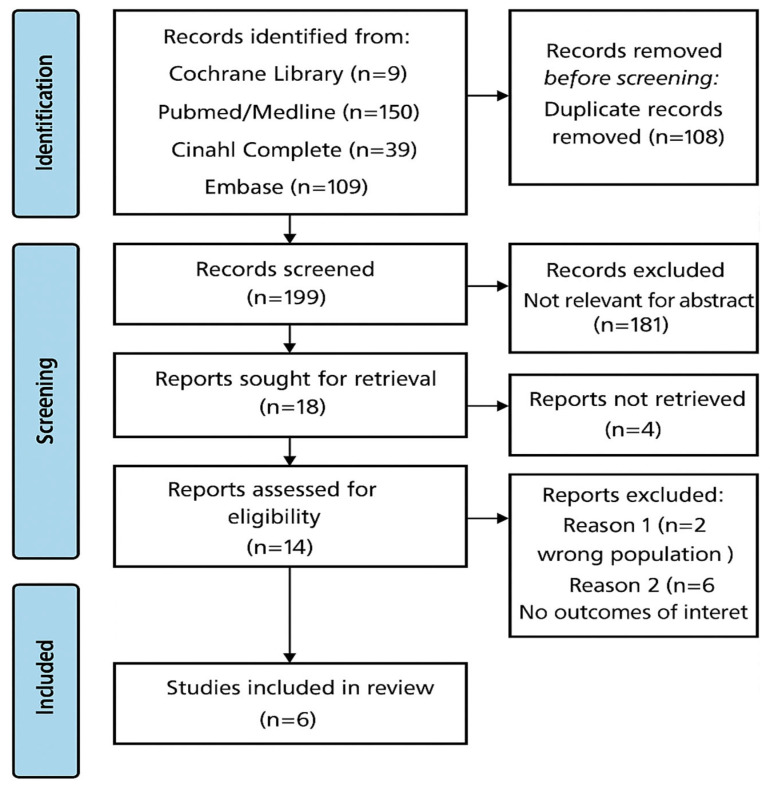
Prisma flow-chart of the process of article inclusion.

A total of 307 articles were identified through database searches: 150 from PubMed-MEDLINE, 9 from the Cochrane Library, 109 from Embase, and 39 from CINAHL. After removing duplicates, 199 potentially relevant documents remained. Following a detailed review of titles and abstracts, 191 articles were excluded as they were either irrelevant to the research question or not appropriate in terms of study design. Ultimately, 6 studies met the inclusion criteria and were included in this umbrella review (Figure 1).

### 3.1. General Characteristics of the Studies Included

Table 1 summarizes the systematic reviews conducted by various authors [54,55,56,57,58], highlighting the number of randomized controlled trials (RCTs) included in each review. The reviews covered publications from 2020 to 2024, with commonly searched databases being PubMed, Cochrane, Embase, and Scopus. The number of RCTs included in these reviews ranged from 7 to 28. Table 2 summarizes the PICO adopted for the single studies included [54,55,56,57,58,59]. Table 3 Summary the Effects of Probiotic Supplementation on Gestational Outcomes with GRADE Assessment.

### 3.2. Overview of Probiotic Effects on GD and Maternal Outcomes

Table 3 provides a summary of the key studies analyzing the role of probiotics in the context of GD. It includes each study’s probiotic strain, dosage, duration of administration, and principal results.

### 3.3. Quality of Included Studies

The risk of bias in individual studies was assessed using the critical appraisal tools provided by the Joanna Briggs Institute. Studies were classified as having a low risk of bias if ≥70% of criteria received positive (“Yes”) responses. All included reviews positively met the criteria related to clarity of the research question, adequacy of inclusion criteria, the comprehensiveness of the search strategy, and appropriateness of resources utilized to identify primary studies [54,55,56,57,58,59]. Methodologies for combining results and minimizing errors during data extraction were deemed sound across all studies. However, one review [56] did not evaluate the risk of publication bias, representing a potential limitation compared to other reviews that included such analysis. Overall, the mean critical appraisal score across all studies was high, at 99% (range: 91–100%), reflecting excellent methodological quality (Table 4).

### 3.4. Efficacy of Probiotics in Preventing GD

The use of probiotics for preventing GD has been extensively studied, with varying results depending on the study designs, populations examined, and probiotic formulations used. Several meta-analyses and systematic reviews have highlighted the potential benefits of multi-strain probiotics, particularly combinations including Lactobacillus rhamnosus GG and Bifidobacterium lactis, in reducing GD risk and improving metabolic outcomes. For instance, a meta-analysis of 12 randomized controlled trials (RCTs) involving 2213 participants reported a significant reduction in GD incidence (RR 0.62; 95% CI), alongside improvements in fasting blood glucose, insulin concentrations, and insulin resistance indices, although no significant effects were observed on oral glucose tolerance test (OGTT) outcomes [57]. Similarly, another meta-analysis of 14 RCTs with 3527 participants confirmed the protective effects of probiotics (RR 0.71; 95% CI: 0.52–0.96; *p* = 0.03), with more pronounced benefits observed in women with a BMI < 26 kg/m^2^ (RR 0.58) and aged under 30 years (RR 0.42). Specifically, probiotic strains such as Lactobacillus acidophilus and Bifidobacterium lactis Bb12 exhibited greater effectiveness in these populations [54]. Mahdizade Ari et al. (2022) supported these findings, highlighting that multi-strain probiotics containing Lactobacillus acidophilus and Bifidobacterium lactis Bb12 significantly improved metabolic parameters related to GD, including blood glucose levels and inflammatory biomarkers, suggesting their potential role as a metabolic control strategy during pregnancy [55]. Similarly, a review of 10 RCTs involving 2921 participants demonstrated a 33% reduction in GD risk (RR 0.67; 95% CI: 0.47–0.95), with the most notable effects attributed to multi-strain probiotics comprised of Lactobacillus rhamnosus, Lactobacillus acidophilus, Bifidobacterium lactis, and Bifidobacterium bifidum [60]. However, not all studies reported significant benefits. A meta-analysis of 17 RCTs with 2550 participants found no substantial reduction in GD risk (OR 0.77; 95% CI: 0.51–1.16; *p* = 0.21), although a modest decrease in fasting blood glucose (−1.01 mg/dL) was observed, which was not clinically relevant [58]. Likewise, a Cochrane review including 7 RCTs with 1647 participants found no conclusive evidence supporting probiotic efficacy in reducing GD risk (RR 0.80; 95% CI: 0.54–1.20). Additionally, this review reported an increased risk of preeclampsia (RR 1.85; 95% CI: 1.04–3.29) associated with generic combinations of Lactobacillus and Bifidobacterium [59].

### 3.5. Improvement of Metabolic Parameters

Probiotic supplementation has been associated with improvements in metabolic parameters relevant to GD risk. Significant reductions in fasting blood glucose, insulin concentrations, and insulin resistance, alongside improvements in sensitivity indices such as HOMA-IR and QUICKI, have been documented in meta-analyses [57]. Additional studies have observed beneficial effects on fasting blood glucose, lipid profiles, and inflammatory markers, reinforcing the positive impact of probiotics on metabolic control (*p* < 0.05) [54]. Conversely, other reviews reported only modest reductions in fasting blood glucose, deemed clinically irrelevant, without significant effects on other metabolic parameters [58].

### 3.6. Effects on Maternal and Neonatal Complications

Evidence regarding the effects of probiotics on maternal and neonatal complications remains inconclusive. Most studies did not report significant improvements in outcomes such as preeclampsia, cesarean delivery rates, or neonatal complications, including macrosomia and prematurity [60]. Interestingly, one review identified a potential increased risk of preeclampsia among women receiving probiotics (RR = 1.85; 95% CI: 1.04–3.29), raising concerns about possible adverse effects [58]. For other outcomes, including cesarean delivery rates (RR = 1.00; 95% CI: 0.86–1.17) and macrosomia (RR = 0.99; 95% CI: 0.72–1.36), no significant differences were observed between probiotic-treated and control groups [59]. Thus, further research is essential to clarify the safety profile and clinical implications of probiotic supplementation during pregnancy.

## 4. Discussion

This umbrella review aims to determine the role of probiotics in preventing GD in healthy pregnant women. Due to the maternal and fetal risks observed in pregnancies complicated by GD, several interventions are planned and implemented to prevent GD and, consequently, improve the health of women and future generations [60]. A study [61], states that most women are unaware of the risk factors associated with the development of GD and regret not having received advice on modifiable factors, such as weight management before pregnancy. Various interventions to prevent GD have been documented in the literature. A low glycemic index diet has been shown to attenuate the increase in insulin resistance observed during pregnancy, thus reducing the risk of GD [62,63,64]. However, this may not be sufficiently effective because individuals vary in their glycemic response to the same foods; for example, one person may experience postprandial hyperglycemia despite consuming low-glycemic-index foods [61]. The inclusion of healthy dietary components, such as fiber, may have an additive effect on reducing maternal glucose concentrations [60,62,65]. Simple interventions based on nutritional supplements, such as myo-inositol, appear to reduce the risk of GD when combined with dietary interventions [60,63,64,66,67,68]. Physical exercise also has a protective role [66,69,70], with a greater reduction in the risk of developing GD observed when an exercise program is maintained throughout pregnancy and chronic care, in line with the principles of a lifestyle medicine view [60,63,71,72,73,74,75,76,77,78,79,80,81]. The use of probiotics for GD prevention is a growing area of interest, with emerging evidence suggesting potential benefits, although it is not uniformly reported across studies. Masulli et al. [58] found that probiotic use did not significantly reduce the incidence of GD (MH-OR 0.77, *p* = 0.21) in a population of 2550 participants with a mean age of 29.4 years. While a slight reduction in fasting plasma glucose (FPG) (−1.01 mg/dL, *p* = 0.02) was observed, this change was not deemed clinically relevant, suggesting that probiotics, in this context, may not have significant preventive effects on GD. In contrast, Zhang et al. [57], in a study conducted on 2213 participants aged 18 to 40 years, reported a significant reduction in GD risk (RR 0.62), along with improvements in metabolic parameters, including fasting glucose, insulin, HOMA-IR, and QUICKI. However, no significant benefit was observed in oral glucose tolerance test (OGTT) outcomes, highlighting potential limitations in the efficacy of probiotics regarding certain key diagnostic indicators of GD. Pakmehr et al. [56], in a larger cohort of 2921 participants, reported a 33% reduction in GD risk (RR 0.67), though no significant improvements in maternal or neonatal complications were noted, thereby limiting the overall clinical relevance of probiotic interventions concerning secondary pregnancy outcomes. Similarly, Mahdizade Ari et al. [55] observed a significant reduction in GD among 4865 pregnant women (*p* < 0.05), with additional metabolic benefits, including improvements in glucose levels, lipids, and inflammatory biomarkers, suggesting the broader metabolic utility of probiotics. The meta-analysis of Li et al. [54], conducted on 3527 participants, confirmed a significant reduction in GD (RR 0.71, *p* = 0.03), with more pronounced effects observed among women with a BMI < 26 kg/m^2^ (RR 0.58) and those under 30 years of age (RR 0.42). These findings underscore the importance of individual factors, such as age and body mass index, in modulating responses to probiotics, suggesting that specific subpopulations may derive greater benefits from such interventions. To enhance the comparability and integration of results across studies, further research with larger sample sizes and standardized criteria for defining GD is needed. Changes during pregnancy may also pose a limitation, as they influence adherence to recommendations due to symptoms such as nausea, taste alterations, and fatigue [60]. Overall, the findings suggest that probiotics may offer promising benefits in preventing GD, particularly in selected populations. However, discrepancies among studies, stemming from differences in probiotic strains, intervention duration, and population characteristics, warrant further investigation to identify the most effective protocols and the subgroups of women who are most responsive to these interventions. In conclusion, while the use of probiotics for the prevention of gestational diabetes is still under study, their potential benefits for treating other chronic conditions are emerging. For instance, probiotics have shown promising results in managing allergic rhinitis by improving symptoms and quality of life [74]. Their role has also been explored in inflammatory bowel disease (IBD), where they can help restore gut microbiota balance; combining standard treatment with probiotics might be an option to achieve remission in active ulcerative colitis patients [75]. Additionally, the impact of probiotics on type 2 diabetes has been reported, contributing to better glycemic control and insulin sensitivity [76].

### 4.1. Limitations

This umbrella review has several limitations. First, the number of relevant meta-analyses and systematic reviews included in the umbrella review is relatively small, although the average critical appraisal score across all studies was 99%, reflecting a high overall methodological quality. Another limitation stems from the fact that the optimal blood glucose concentration for diagnosing GD remains controversial, and the outcome was measured using various indicators, such as fasting blood glucose (FBG), insulin concentration, insulin resistance and sensitivity indices, homeostasis model assessment of insulin resistance (HOMA-IR), QUICKI, and OGTT. Furthermore, the administration of the intervention in the studies considered in each meta-analysis and systematic review varied both in terms of duration and in the type of probiotic used. Finally, no subgroup analyses were conducted based on factors such as ethnical difference, age, weight, or the pregnant woman’s condition, which could introduce bias. Due to the heterogeneity in outcome measures, interventions, and populations across the included meta-analyses and systematic reviews, as well as the limited number of studies, we did not perform a new quantitative synthesis or produce a forest plot in this umbrella review.

### 4.2. Perspective for Clinical Practice

This underscores the critical importance of implementing effective preventive strategies in women planning pregnancy or already pregnant [77]. Probiotic supplementation represents a promising, low-cost, and non-pharmacological intervention that aligns with the broader public health goals to reduce the possible collateral effect in a chronic disease burden [78,79,80]. Beyond gestational diabetes, probiotics have shown beneficial effects in the management of metabolic parameters and inflammatory processes associated with chronic conditions such as obesity, type 2 diabetes, and cardiovascular disease [81,82,83,84,85,86,87]. Their integration into maternal health strategies may offer a dual benefit, supporting both short- and long-term health outcomes for mothers and offspring. In addition, the advancement of digital health technologies offers new opportunities to personalize and scale preventive strategies in chronic care in general and GD in particular [88,89,90,91,92,93]. Mobile applications, remote monitoring, and telehealth platforms could facilitate the delivery of, and adherence to, probiotic-based interventions as part of integrated antenatal care and chronic disease prevention programs [94,95]. From a public health perspective, embedding these tools into structured care pathways could enhance the reach and impact of early preventive actions, particularly among high-risk populations [96,97,98]. Nevertheless, clinical recommendations should continue to be evidence-based and individualized, while ongoing research refines our understanding of the most effective probiotic strains, dosages, and delivery models.

## 5. Conclusions

GD remains a pressing global health issue with significant consequences for maternal and fetal outcomes. This umbrella review synthesized current evidence on the role of probiotics in GD prevention, highlighting promising, though not yet conclusive, results. Benefits were most evident in specific populations, particularly younger women with lower BMI, and when probiotics were used in conjunction with healthy lifestyle behaviors. Among the probiotic strains evaluated, multi-strain formulations including Lactobacillus rhamnosus GG, Lactobacillus acidophilus, Bifidobacterium lactis, and Bifidobacterium bifidum showed the most potential in improving metabolic parameters and reducing GD risk. However, inconsistencies across studies and considerable heterogeneity limit the generalizability of these findings. Further high-quality, strain-specific research is essential to determine optimal treatment protocols and to identify subgroups most likely to benefit from probiotic interventions. These insights are crucial for developing targeted, evidence-based prevention strategies in at-risk pregnant populations.

## Figures and Tables

**Table 1:** Data Extraction of Relevant Studies.

First Author	Publication Year	Studies (n)	Study Design Included
Li et al. [54]	2024	14	RCTs
Mahdizade Ari et al. [55]	2022	28	RCTs
Pakmehr et al. [56]	2022	10	RCTs
Zhang et al. [57]	2022	12	RCTs
Davidson et al. [59]	2021	7	RCTs
Masulli et al. [58]	2020	17	RCTs

Legend: RCTs: Randomized Controlled Trials.

**Table 2:** PICO Summary of the Included Systematic Reviews and Meta-Analyses.

Author (Year)	Population	Intervention	Comparator	Outcomes Reported
Li et al. (2024) [54]	3527 pregnant women, aged 27–34 years, BMI 21–39 kg/m^2^, mostly in early pregnancy; 14 RCTs conducted in 10 countries	Probiotic supplementation (various strains including *L. rhamnosus*, *B. lactis*, *S. thermophilus*), 1–50 × 10^9^ CFU/day, started during 1st or 2nd trimester	Placebo (12 RCTs) or no additional treatment (2 RCTs)	Incidence of GDM; subgroup analyses by BMI and age
Mahdizade Ari et al. (2022) [55]	6014 pregnant women with or without GDM; 25 RCTs (various countries)	Oral probiotic supplementation (various strains; mean duration 4–12 weeks)	Placebo	Incidence of GDM; fasting plasma glucose (FPG); insulin; HOMA-IR; neonatal outcomes (birth weight, prematurity)
Zhang et al. (2022) [57]	2213 pregnant women without diabetes; 12 RCTs (China, Iran, Finland, Australia, others)	Probiotic supplementation (various strains; 4–12 weeks duration)	Placebo	GDM incidence; fasting blood glucose (FBG); insulin; HOMA-IR; QUICKI; 1 h and 2 h OGTT
Masulli et al. (2020) [58]	2968 pregnant women with or without GDM, mean age ~29.4 years, mean BMI ~28.5 kg/m^2^; 17 RCTs (various countries)	Probiotic supplementation (various strains, mostly *L. rhamnosus*, *B. lactis*, *L. salivarius*, etc.), duration ~11.5 weeks	Placebo	Incidence of GDM; fasting plasma glucose (FPG); fasting insulin; HOMA index; maternal and fetal outcomes
Pakmehr et al. (2022) [56]	2921 healthy pregnant women without previously diagnosed glucose disturbances; 10 RCTs	Probiotic supplementation (various strains, especially multi-strain; dose, timing and duration varied)	Placebo	Primary: Incidence of GDM. Secondary: maternal and infant outcomes (e.g., preeclampsia, cesarean, macrosomia, NICU, prematurity)
Davidson et al. (2021) [59]	Pregnant women without pre-existing diabetes; studies from Australia, Finland, Iran, Ireland, and New Zealand; 7 RCTs	Probiotic supplementation (various strains, doses, forms, mostly capsules; initiated before 20 weeks of gestation)	Placebo	GDM diagnosis; pre-eclampsia; hypertensive disorders; cesarean section; gestational weight gain; large-for-gestational-age infants; neonatal outcomes

Legend: BMI = Body Mass Index; CFU = Colony-Forming Units; FPG = Fasting Plasma Glucose; FBG = Fasting Blood Glucose; GDM = Gestational Diabetes Mellitus; HOMA-IR = Homeostasis Model Assessment of Insulin Resistance; NICU = Neonatal Intensive Care Unit; OGTT = Oral Glucose Tolerance Test; QUICKI = Quantitative Insulin Sensitivity Check Index; RCT = Randomized Controlled Trial.

**Table 3:** Summary of Findings: Effects of Probiotic Supplementation on Gestational Outcomes (GRADE Assessment).

Author (Year)	No. RCTs (n Participants)	Outcome	Relative Effect (RR/OR, 95% CI)	Absolute Effect	I^2^	τ^2^	GRADE
Li et al. (2024) [54]	14 RCTs (3527 women)	GDM incidence	RR: 0.71 (95% CI: 0.52–0.96)	ARR: –5.3%; Probiotics 13.1%, Placebo 18.4%	73%	NR	⬤⬤⬤◯ Moderate (inconsistency)
Mahdizade Ari et al. (2022) [55]	25 RCTs (6014 women)	GDM incidence	RR: 0.73 (95% CI: 0.58–0.91)	ARR: –5.8%; Event rates not reported	65%	0.03	⬤⬤⬤◯ Moderate (heterogeneity)
	25 RCTs (6014 women)	FPG	MD: –3.10 mg/dL (95% CI: –5.21 to –0.98)	–	87%	4.7	⬤⬤◯◯ Low (inconsistency, imprecision)
Zhang et al. (2022) [57]	12 RCTs (2213 women)	GDM incidence	RR: 0.62 (95% CI: 0.39–0.99)	ARR: –4.9%; Event rates not reported	58%	0.01	⬤⬤⬤◯ Moderate (some inconsistency)
	12 RCTs (2213 women)	FBG	MD: –2.52 mg/dL (95% CI: –4.61 to –0.44)	–	72%	2.1	⬤⬤⬤◯ Moderate (imprecision)
Masulli et al. (2020) [58]	17 RCTs (2968 women)	GDM incidence	OR: 0.77 (95% CI: 0.51–1.16)	Not statistically significant	62%	NR	⬤⬤⬤◯ Moderate (wide CI)
	15 RCTs (n not specified)	FPG	MD: –1.05 mg/dL (95% CI: –1.95 to –0.16)	Minimal, not clinically relevant	45%	NR	⬤⬤⬤◯ Moderate (precision)
Pakmehr et al. (2022) [56]	10 RCTs (2921 women)	GDM incidence	RR: 0.67 (95% CI: 0.47–0.95)	ARR: –4.5%; Probiotics 8.4%, Placebo 12.9%	NR	NR	⬤⬤⬤◯ Moderate (data limited)
Davidson et al. (2021) [59]	6 RCTs (1440 women)	GDM incidence	RR: 0.80 (95% CI: 0.54–1.20)	Not reported	NR	NR	⬤⬤◯◯ Low (inconsistency, imprecision)
	4 RCTs (955 women)	Pre-eclampsia	RR: 1.85 (95% CI: 1.04–3.29)	–	NR	NR	⬤⬤⬤⬤ High (robust effect)
	6 RCTs (1520 women)	Caesarean section	RR: 1.00 (95% CI: 0.86–1.17)	–	NR	NR	⬤⬤⬤⬤ High (no effect)
	4 RCTs (853 women)	Weight gain in pregnancy	MD: 0.30 kg (95% CI: –0.67 to 1.26)	–	NR	NR	⬤⬤⬤◯ Moderate (inconsistency)
	4 RCTs (919 women)	Large-for-gestational age	RR: 0.99 (95% CI: 0.72–1.36)	–	NR	NR	⬤⬤⬤◯ Moderate (imprecision)
	3 RCTs (709 women)	Perinatal mortality	RR: 0.33 (95% CI: 0.01–8.02)	–	NR	NR	⬤⬤◯◯ Low (very serious imprecision)
	2 RCTs (623 women)	Morbidity composite	RR: 0.69 (95% CI: 0.36–1.35)	–	NR	NR	⬤⬤◯◯ Low (very serious imprecision)
	2 RCTs (586 women)	Neonatal hypoglycaemia	RR: 1.15 (95% CI: 0.69–1.92)	–	NR	NR	⬤⬤◯◯ Low (inconsistency, imprecision)
	2 RCTs (320 women)	Neonatal adiposity	MD: −0.04 kg & −0.10%	Not pooled	–	–	⬤⬤◯◯ Low (imprecision)

Legend. ARR = Absolute Risk Reduction; CI = Confidence Interval; FBG = Fasting Blood Glucose; FPG = Fasting Plasma Glucose; GDM = Gestational Diabetes Mellitus; MD = Mean Difference; NR = Not Reported; OR = Odds Ratio; RCT = Randomized Controlled Trial; RR = Risk Ratio; τ^2^ = Between-study variance; I^2^ = Heterogeneity index. ⬤ = solid circle, criterion fully met; ◯ = empty circle, criterion not met or uncertain.

**Table 4:** Critical Appraisal of the Included Studies.

	Items												
Study	1	2	3	4	5	6	7	8	9	10	11	Include	Score (Mean)
Li et al., 2024 [54]	Y	Y	Y	Y	Y	Y	Y	Y	Y	Y	Y	Y	100%
Mahdizade Ari et al, 2022 [55]	Y	Y	Y	Y	Y	Y	Y	Y	Y	Y	Y	Y	100%
Zhang et al., 2022 [57]	Y	Y	Y	Y	Y	Y	Y	Y	Y	Y	Y	Y	100%
Masulli et al., 2020 [58]	Y	Y	Y	Y	Y	Y	Y	Y	Y	Y	Y	Y	100%
Pakmehr et al., 2022 [56]	Y	Y	Y	Y	Y	Y	Y	Y	Y	Y	Y	Y	100%
Davidson et al., 2021 [59]	Y	Y	Y	Y	Y	Y	Y	Y	N	Y	Y	Y	91%

Legend: Y = Yes; N = No; 1 = Were the criteria for inclusion in the sample clearly defined? 2 = Were the study subjects and the setting described in detail? 3 = Was the exposure measured in a valid and reliable way? 4 = Were objective, standard criteria used for measurement of the condition? 5 = Were confounding factors identified? 6 = Were strategies to deal with confounding factors stated? 7 = Were the outcomes measured in a valid and reliable way? 8 = Was appropriate statistical analysis used?? 9 = Were the response rate and the reasons for non-response reported? 10 = Was appropriate follow-up (if applicable) conducted and described? 11 = Was ethical approval or informed consent reported?

## Data Availability

The data supporting this research are available upon request from the corresponding author for data protection reasons.

## Supplementary Materials

The following supporting information can be downloaded at: https://www.mdpi.com/article/10.3390/jcm14145168/s1. Supplementary File S1: PRIOR 2022 Checklist; Supplementary File S2: Search Strategy.
